# Supplementary material for: The interactome of intact mitochondria by cross-linking mass spectrometry provides evidence for coexisting respiratory supercomplexes
Source: Mol Cell Proteomics. 2017 Dec 8;17(2):216–32. doi: 10.1074/mcp.RA117.000470 (PMC5795388; doi:10.1074/mcp.RA117.000470)
Supplement: Supplemental Data [file supp_RA117.000470_133922_0_supp_23978_fzffwf.zip › spectra_annotation/mito_spectra_annotation/71-1-10-1-10-1.pdf]

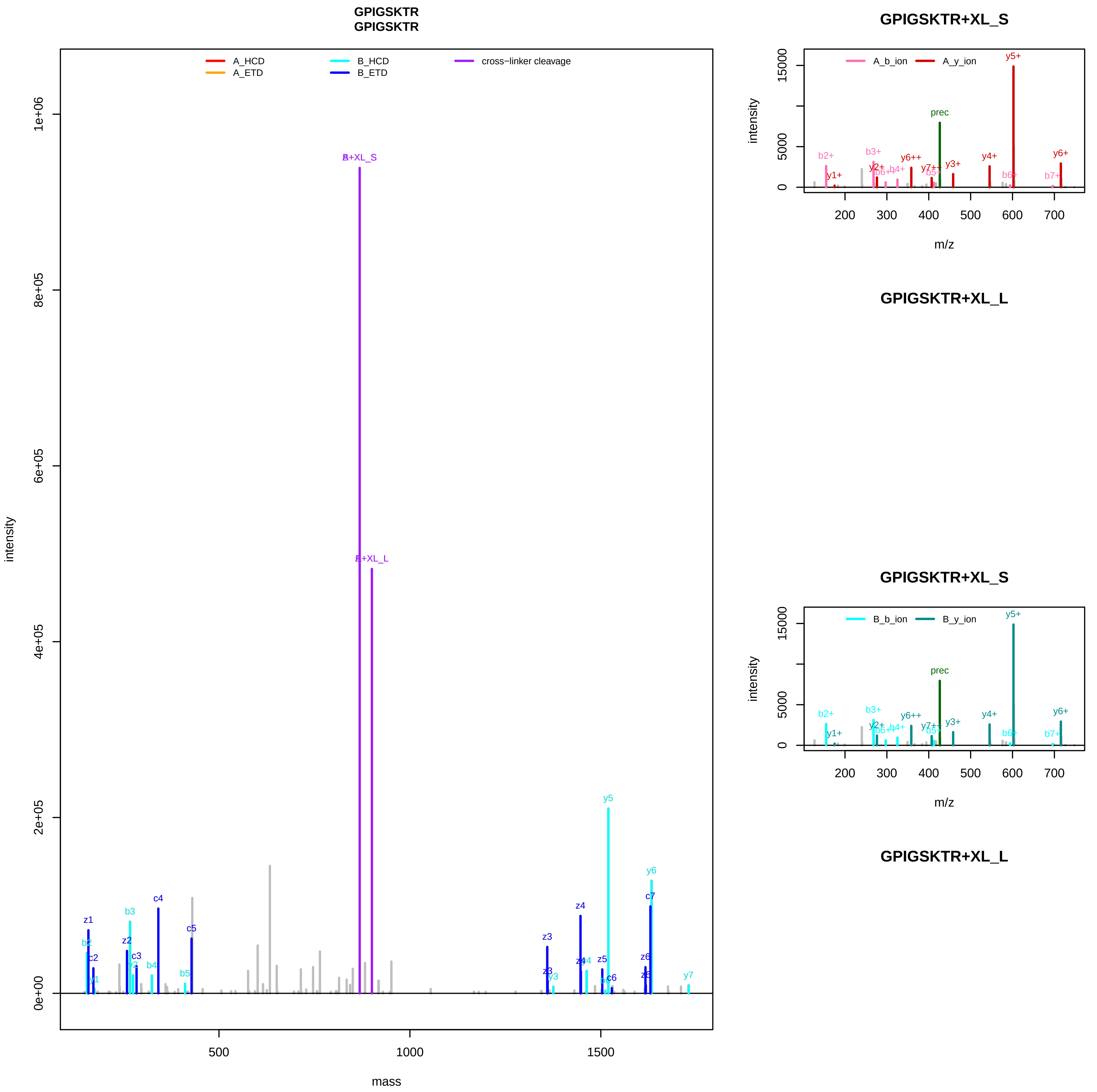
GPIGSKTR
GPIGSKTR
A_HCD
A_ETD
B_HCD
B_ETD
cross−linker cleavage
intensity
mass
0e+00
2e+05
4e+05
6e+05
8e+05
1e+06
500
1000
1500
B+XL_S
B+XL_L
z1
b2
c2
y1
z2
b3
c3
c4
b4
c5
b5
z3
z4
z5
c6
y5
y6
c7
z6
y7
GPIGSKTR+XL_S
A_b_ion
A_y_ion
prec
y5+
b2+
y1+
b3+
y2+
y6++
y7++
y3+
y4+
y6+
b6+
b7+
intensity
m/z
0
5000
15000
200
300
400
500
600
700
GPIGSKTR+XL_L
GPIGSKTR+XL_S
B_b_ion
B_y_ion
prec
y5+
b2+
y1+
b3+
y2+
y6++
y7++
y3+
y4+
y6+
b6+
b7+
intensity
m/z
0
5000
15000
200
300
400
500
600
700
GPIGSKTR+XL_L
